# Dominant and Modifiable Risk Factors for Dementia in Sub-Saharan Africa: A Systematic Review and Meta-Analysis

**DOI:** 10.3389/fneur.2021.627761

**Published:** 2021-03-25

**Authors:** Akin Ojagbemi, Akinkunmi Paul Okekunle, Opeyemi Babatunde

**Affiliations:** ^1^Department of Psychiatry, College of Medicine, University of Ibadan, Ibadan, Nigeria; ^2^Department of Epidemiology and Medical Statistics, College of Medicine, University of Ibadan, Ibadan, Nigeria; ^3^Department of Food and Nutrition, College of Human Ecology, Seoul National University, Seoul, South Korea; ^4^School of Medicine Primary Care Center Versus Arthritis Keele University, Staffordshire, United Kingdom

**Keywords:** low-and middle-income countries, Sub-Saharan Africa, dementia prevalence, dementia incidence, risk factors

## Abstract

**Background:** Sub-Saharan Africa (SSA) is projected to have a rapid increase in the number of people living with dementia by 2050. Yet, there is currently no robust evidence on the risk factors for dementia in the sub-region that could inform context specific interventions.

**Methods:** We conducted a systematic review and meta-analysis of observational studies to determine the dominant and modifiable risk factors for dementia in SSA. We searched MEDLINE, EMBASE, PsychINFO, and African Journals Online using keywords for dementia and Alzheimer's disease as well as the.mp operator for all 47 SSA countries or regions. We included peer-reviewed original studies with epidemiological designs, conducted random effect meta-analysis and determined the dominant and modifiable risk factors for dementia using the inverse of variance method.

**Results:** A total of 44 studies out of 2,848 met criteria for syntheses. The pooled annual incidence of dementia from 5,200 cohort risk years was 2.0% [(95% Confidence Interval (CI) = 1.0–4.0%)]. The pooled prevalence was 5.0% (95% CI = 2.0–7.0%). Older age was the dominant risk factor for both prevalent [(Standard error (S.E = 0.3, weight = 25.2%)] and incident dementia (S.E = 0.02, weight = 95.8%), while low educational attainment (S.E = 0.19, weight = 32.6%) and poor predementia cognitive functioning at baseline (S.E = 0.2, weight = 20.5%) were the best ranked modifiable risk factor for incident dementia.

**Conclusion:** Low formal educational attainment which, in SSA, may represent a stable index of low socioeconomic position and health disadvantage over the life course, was the most prominent modifiable risk factor for incident dementia. Findings have implications for deliberate policies targeted at access to education across the life course as a primary prevention strategy against dementia in SSA.

## Introduction

Sub-Saharan Africa (SSA) is set to have one of the largest increases in the population of older people worldwide (1), and by 2050, approximately 161 million persons who are 60 years or older will be residents of the sub-region (2). The prevalence and incidence of dementia increases with age (2, 3). Yet, there is currently no robust evidence on the risk factors for dementia in SSA that could inform context specific interventions.

In our previous study (3), we found a 4% pooled prevalence of clinically diagnosed dementia from an overall sample 6964 community-dwellers who were 60 years or older. The previous review (3), and others conducted by Alzheimer's Disease International (2, 4), had searched databases until May 2016, and as there were few published information on incidence of dementia at the time, the evidence was limited to cross-sectional prevalence of dementia in SSA.

In the succeeding four and half years, the literature on the epidemiology of dementia in SSA has been boosted by the publication of new data which have provided valuable additional information. In particular, longitudinal follow-up data (5–9) may serve to build on evidence provided by cross-sectional surveys of dementia in SSA. Such data should allow for an investigation of the links between cross-sectionally identified risk predictors (3) and subsequent onset of dementia. Longitudinal studies may also provide evidence for the relative importance of each modifiable risk factor for incident dementia, information required for the prioritization of primary prevention targets within limited resource contexts of SSA.

The aim of the present study was to conduct a systematic review and meta-analyses of epidemiological studies on dementia in SSA. Specifically, in addition to new information on the annual incidence of dementia in SSA, we aimed to identify key modifiable risk factors for onset of dementia among elders in SSA communities. Estimates of general hospital frequency, community prevalence, as well as their correlates was also profiled.

## Methods

This review followed conventional recommendations for the methodology and reporting of systematic reviews as described in the guidelines of the National Institute of health and Care Excellence (NICE) and Preferred Reporting Items for Systematic reviews and Meta-analyses (PRISMA) (10, 11). We registered our study protocol in the International prospective register of systematic reviews (#CRD42021214843).

### Search Strategy

An initial search of the African Journals Online (AJOL) database was conducted on 15th September 2020. This was followed by a search of the MEDLINE, PsychINFO, and Embase databases. For these searches, a facet analyses was constructed using appropriate modifications of the PICO framework (10). The following keywords identified according to facets in the modified PICO were searched with the “explode” operator to retrieve other similar terms: dementia or “Alzheimer's disease”, AND epidemiology OR frequency OR prevalence OR incidence OR factors OR “risk factors” OR “associated factors” (Box 1). We next combined a search of each of the 47 SSA countries or regions by name using the.mp. operator. A second stage consisting of hand searching of the reference list of relevant articles retrieved from the databases was also implemented. Limits on language and publication dates were not imposed in conducting the searches.

Box 1 **MEDLINE search terms using the Pubmed interphase.**

[(dementia OR “Alzheimer's disease”) AND (epidemiology OR frequency OR prevalence OR incidence OR factors OR “risk factors” OR “associated factors' OR outcome OR mortality)] AND (Angola OR Benin OR Botswana OR “Burkina Faso” OR Burundi OR Cameroun OR “Central African Republic” OR Chad OR Congo OR “Cote d'Ivoire” OR Eritrea OR Ethiopia OR Gabon OR Gambia OR Ghana OR Guinea OR Guinea-Bissau OR Kenya OR Lesotho OR Liberia OR Madagascar OR Malawi OR Mali OR Mauritania OR Mauritius OR Mozambique OR Namibia OR Niger OR Nigeria OR Rwanda OR Senegal OR “Sierra Leone” OR Somalia OR “South Africa” OR “United Republic of Tanzania” OR Togo OR Uganda OR Zaire OR Zambia OR Zimbabwe OR “Sub Saharan Africa” OR sub-Saharan Africa) AND ((y_5[Filter]) AND (humans[Filter])).

#### Inclusion Criteria

Studies were included if; (1) they investigated epidemiological phenomena such as frequencies, prevalence, incidence, risk or associated factors, (2) they included participants with any type of dementia regardless of setting, method of ascertainment or diagnosis, (3) descriptive and analytical cross-sectional studies, prospective and retrospective cohort studies, case control studies, randomized controlled trials, non-randomized controlled trials, as well as quasi-experimental studies.

#### Exclusion Criteria

We excluded the following types of studies, (1) review papers, case series, individual case reports, expert opinions, discussion papers, and position papers; and (2) studies focusing solely on qualitative data.

### Study Assessments and Data Extraction

Study assessment for inclusion and exclusion criteria as well as subsequent data extraction was conducted by two independent assessors (AO and APO) based on the descriptions in the original article. The following information were extracted from each included study: first author name, publication year, diagnostic criteria, sample size, average age at baseline, the proportion of females, hospital frequency, community prevalence, average follow-up time, cohort risk years, annual incidence, adjusted relative risks/hazard ratios/odds ratios (RRs/HRs/ORs) with their 95% confidence intervals (Cis), the number of participants and cases for each exposure level and the main covariates of Alzheimer's disease or dementia. Only studies with usable data and appropriate analytical techniques were combined in meta-analyses.

### Statistical Methods

Meta-analysis was conducted using estimates reported in the original articles. The 95% C.I of each estimate was used to generate standard errors (S.E) using methodologies developed by the Cochrane collaboration (12). The summary estimates together with their S.E are presented.

As heterogeneity was expected due to differences in the type of dementia assessments (clinical diagnostic criteria or rating scales) as well as setting of studies, a random effect meta-analysis model was chosen. To reduce the extent of methodological heterogeneity, we combined studies with similar diagnostic procedures in the same subgroup meta-analysis. To determine the extent of statistical heterogeneity, we estimated the percentage of total variation in estimates reported across studies that is due to heterogeneity, rather than chance. This was computed using the *I*^2^ test. In the present study, values of *I*^2^ > 50% were chosen as evidence of statistical heterogeneity (13). Publication bias was assessed with the aid of a funnel plot.

For the objective of investigating the most important factors associated with dementia by rank, we used the log of effect ratios and the corresponding S.E of the associations. The inverse of variance method was used for weighting. All analyses were conducted using the Cochrane review manager (Revman) version 5.3 software (14).

## Results

**Figure 1:**
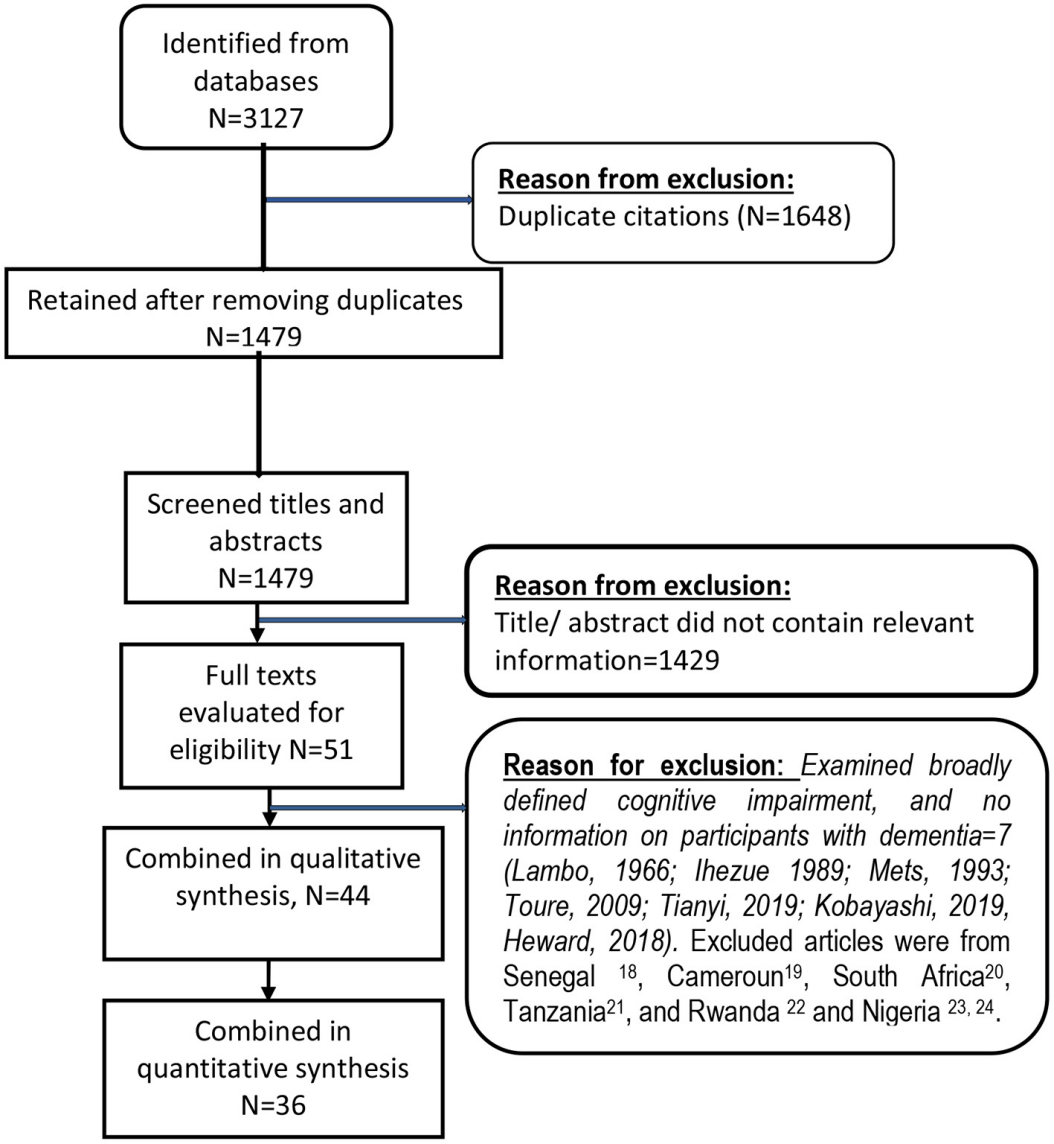
Flow chart showing details of included and excluded studies.

The combined database and hand searches identified a total of 3127 records. After removing duplicates in the databases (*N* = 1,648 articles), 1,479 titles and abstracts were screened. From these, 51 articles with information relevant to the review were retrieved and their full text evaluated. After reading through the texts, 7 articles were further excluded because they examined broadly defined cognitive impairment and did not provide information about participants with dementia (Figure 1). Of the Seven excluded articles, one each was from Senegal (15), Cameroun (16), South Africa (17), Tanzania (18), and Rwanda (19), while the remaining two were from Nigeria (20, 21).

Studies included were published between February 1992 and December 2019. Over 60% of identified studies were publications of data from 6 major research programs (Indianapolis Ibadan Dementia Project, Epidemiology of Dementia in Central Africa-EDAC-, Epidemiology of Dementia in Central Africa-EPIDEMCA-, EPIDEMCA Follow-up, Ibadan Study of Aging, Kilinmajaro cohort from the Hai District of rural Tanzania). *S*tudies represented all regions in SSA: West, East, Central, and Southern Africa. However, about 45.2% of identified studies were from one country, Nigeria.

### Types of Study Settings and Designs

Eight studies (22–29) relied on hospital records (Table 1). Also included in Table 1 are two report of cognitive examination conducted on older people living in residential or nursing homes in Nigeria (30) and South Africa (32), respectively. One study was conducted in a Senegalese primary health center (PHC) (31) (Table 1). The majority (64.5%) of identified studies were community based, including reports of eight prospective longitudinal observations of between 2- and 10-years duration (5–9, 44–46).

**Table 1:** Characteristics of studies of prevalence and incidence of dementia in sub-Saharan Africa.

**References**	**Country**	**Setting**	**Definition of dementia**	**Sample size**	**Female%**	**Age, mean (SD)**	**Frequency (%)**
**Hospital or nursing home studies**							
Ogunniyi et al. (22)	Nigeria	General hospital medical In-patients	ICD 9 criteria	37	24.3	67 (9.0)	0.6
Osuntokun et al. (23)	Nigeria	Autopsy	Histological hallmarks	198	46.0	40-85	0
Baiyewu et al. (30)	Nigeria	Nursing homes	DSM III-R criteria	23	47.8	78.7 (8.6)	48
Napon et al. (27)	Burkina Faso	General hospital In- and outpatient	DSM IV	15,817	33.3	62.2^f^	0.5
Siddiqi et al. (25)	Zambia	General hospital Out/Inpatient	Clinician best judgment	811	52.2	39^f^ (15–80)^g^	2.9/4.0 Out/Inpatient
Toure et al. (31)	Senegal	Primary care center for the elderly	Clinician best judgment	507	<50	72.4 (5.25)	8.87
Amoo et al. (29)	Nigeria	Neuropsychiatric hospital In- and out-patient	ICD 10 criteria	240,294	52.8	70.1 (9.8)	0.05
Ramlall et al. (32)	South Africa	Residential homes for the elderly	DSM IV-TR	140	69.3	75.2 (8.9)	7.9
Ouango et al. (24)	Burkina Faso	General hospital In- and outpatients	Clinician best judgment	7,974	40.2	49–90^g^	1.9
Callixte et al. (26)	Cameroun	Neurology Outpatient	ICD 10 criteria	912	50.8	68.8 (7.2)	12.4
Paddick et al. (28)	Tanzania	General hospital medical In-patients	DSM IV	507	44.4	75^a^ (67–81)^g^	18.7
**References**	**Country/Location**	**Definition of dementia**	**Sample size**	**Female (%)**		**Age, mean (SD)**	**Prevalence (%)**
**Community based cross-sectional surveys**							
**Clinically diagnosed dementia**							
Osuntokun et al. (33)	Nigeria (Idikan)	DSM III-R	930	61.2		40–85	0
Hendrie et al. (6)^a^	Nigeria (Idikan)	ICD 10/DSM III-R	2,494	71.4		81.0 (9.9)	2.29
Guerchet et al. (34)	Benin (Djidja)	DSM-IV	502	57.0		76.1 (9.4)	2.6
Yusuf et al. (35)	Nigeria (Zaria)	ICD 10/DSM IV	322	60.2		75.5 (9.4)	2.8
Guerchet et al. (36)^b^	CAR (Bangui)	DSM IV/Alzheimer's Association	496	55.6		77.4 (7.3)	8.1
Guerchet et al. (36)^b^	Congo (Brazzaville)	DSM IV/Alzheimer's Association	520	40.9		74.7 (6.7)	6.7
Paddick et al. (37)^c^	Tanzania (Hai)	DSM IV	1,198	56.2		≥70^e^	6.4
Ogunniyi et al. (38)	Nigeria (Lalupon)	DSM IV/Alzheimer's Association	613	69.7		72.9 (8.9)	2.9
Guerchet et al. (4)^d^	CAR (Nola)	DSM IV	475	N/A		N/A	8.4
Guerchet et al. (4)^d^	Congo (Gamboma)	DSM IV	529	N/A		N/A	5.7
**Rating scales defined dementia**							
Ochayi et al. (39)	Nigeria (Jos)	CSID	280	89.0		77.2 (9.7)	6.4
Gureje et al. (40)	Nigeria (West/Central regions)	10 Words list learning/Delayed recall test	2,152	53.8		74.5 (8.4)	10.1
Paraiso et al. (41)	Benin	CSID/Five word test	1,139	54.1		73.4 (7.2)	3.7
Van der Poel and Heyns (42)	South Africa (Muangang)	CSID, Geriatric mental state, 10 words list	200	N/A		N/A	6
de Jager et al. (43)	South Africa (Eastern Cape)	CSID	1,382	68.6		71.3 (8.3)	11
**References**	**Country/location**	**Definition of dementia**	**Years of observation (Cohort risk)**	**Female %**		**Age, mean (SD)**	**Annual Incidence (%)**
**Community-based longitudinal observation for incident dementia**							
Hendrie et al. (6)	Nigeria (Idikan)	CERAD Neuropsychological battery/ICD 10 and DSM III-R criteria	5 (2459)	58.9		77.9 (8.0)	1.4
Gureje et al. (7)	Nigeria (West and North-central regions)	10-word listing, delayed recall tests and CHIF	3 (1225)	40.4		74.5 (8.4)	2.2
Samba et al. (8)	Rural and Urban Congo	DSM IV	2 (847)	≈59.7		73.0 (6.6)	2.38
Ojagbemi et al. (5)	West and North-central Nigeria	10-word listing, Delayed recall tests and CHIF	5 (1894)	40.2		74.4 (8.8)	2.1
Gao et al. (9)	Western Nigeria	CERAD Neuropsychological battery/ICD 10 and DSM III-R criteria	N/A (1895)	67		75.7 (5.4)	1.4

*Not included in the table are thirteen duplicate publications from six major research programs (Indianapolis Ibadan Dementia Project, Epidemiology of Dementia in Central Africa-EDAC-, Epidemiology of Dementia in Central Africa-EPIDEMCA-, EPIDEMCA Follow-up, Ibadan Study of Aging, Kilinmajaro cohort from the Hai District of rural Tanzania); SD, Standard deviation; DSM, Diagnostic and Statistical Manual of Mental disorders; III-R, Text revision of 3rd edition; IV, 4th Edition; IIDP, Indianapolis Ibadan Dementia Project; ICD 10, 10th Revision of the International Classification of Diseases; EDAC, Epidemiology of Dementia in Central Africa; CAR, Central African Republic; EPIDEMCA, Epidemiology of Dementia in Central Africa; CSID, Community Screening Instrument for Dementia*.

a *Reported in four studies with 21.6% also meeting 10/66 dementia research group criteria*.

b *Reported in five studies*.

c *Reported in three studies*.

d *Reported in four studies*.

e *All participants were 70 years or older*.

f *Median*.

g *Range*.

### Ascertainment of Dementia

The majority of included studies used a two staged procedure and made formal clinical diagnoses of dementia according to codified criteria (47, 48). However, two hospital based (24, 25) and one PHC study (31) relied on clinicians' best judgement of dementia. Also, seven community based cross-sectional surveys (5, 7, 39–41, 43, 49) used rating scales, including the community screening instrument for dementia, ten words list and delayed recall test, five words test and geriatric mental state examination.

### Meta-Analysis

A total of 36 studies provided usable data for quantitative syntheses (Figure 1).

#### Prevalence and Incidence of Dementia

**Figure 2:**
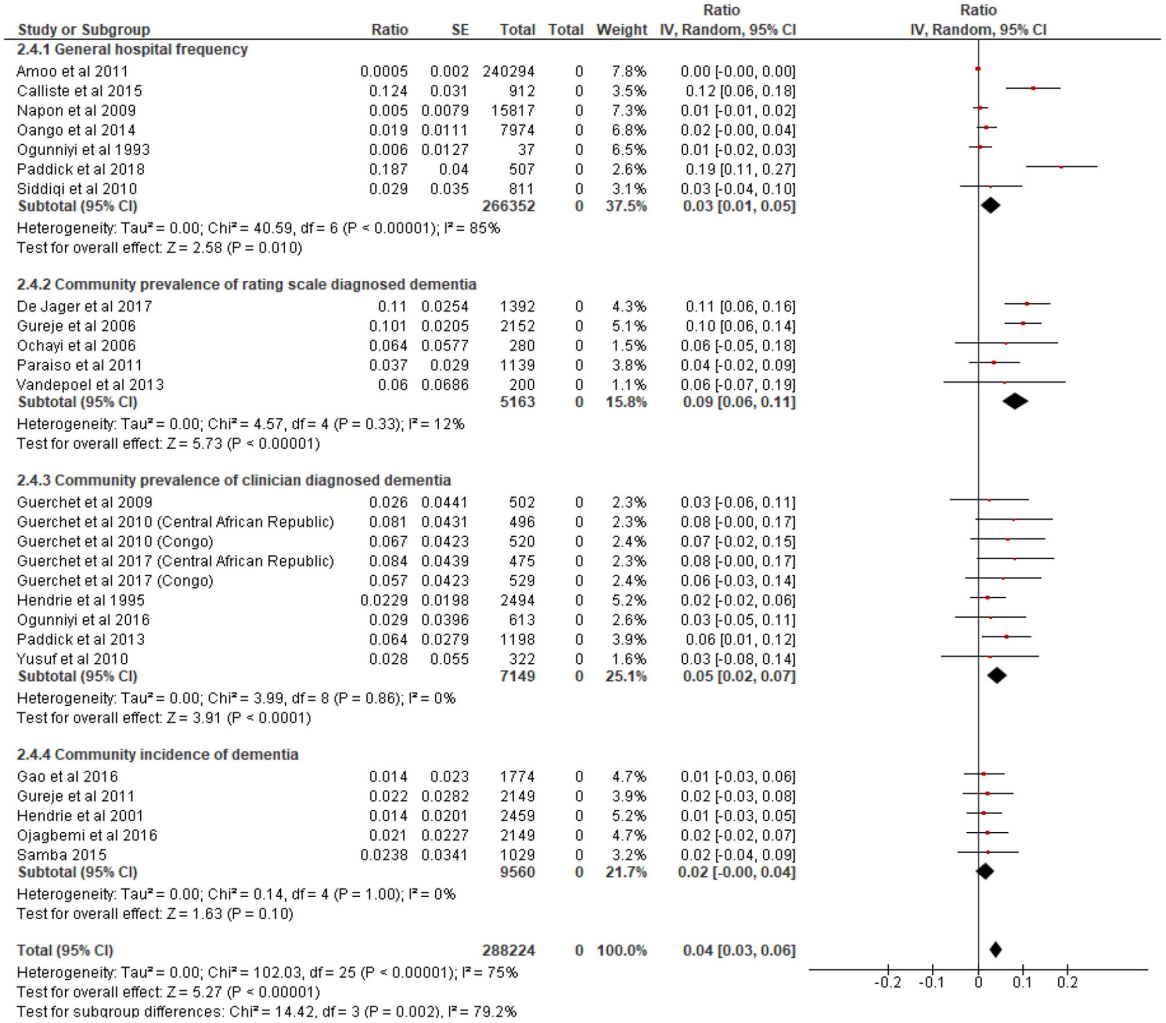
Forest plot showing hospital frequency, community prevalence and incidence of dementia in sub-Saharan Africa. We centered the display of the estimates on the point of zero for better illustration.

Figure 2 presents a forest plot showing the prevalence and incidence of dementia in SSA. Pooled data from seven studies including 266, 352 patients generated a frequency of 3.0% (95% C.I = 1.0–5.0%) for dementia in hospital settings. There was an indication of statistical heterogeneity in this estimate (*I*^2^ = 85%, *p* < 0.001). Heterogeneity was investigated and found to be due to rate outliers of 12.4% (26) and 18.7% (28) reported in two studies. A community prevalence of 9.0% (95% C.I = 6.0–11.0%) was estimated from five studies including 5,153 persons who underwent rating scales assessments for dementia. The pooled community prevalence of clinically diagnosed dementia from ten studies including 8,069 participants was 5.0% (95% C.I = 2.0–7.0%). The pooled annual incidence of dementia from five studies with a total of 5,200 cohort risk years was 2.0% (1.0–4.0%).

#### Risk Factors for Dementia

**Figure 3:**
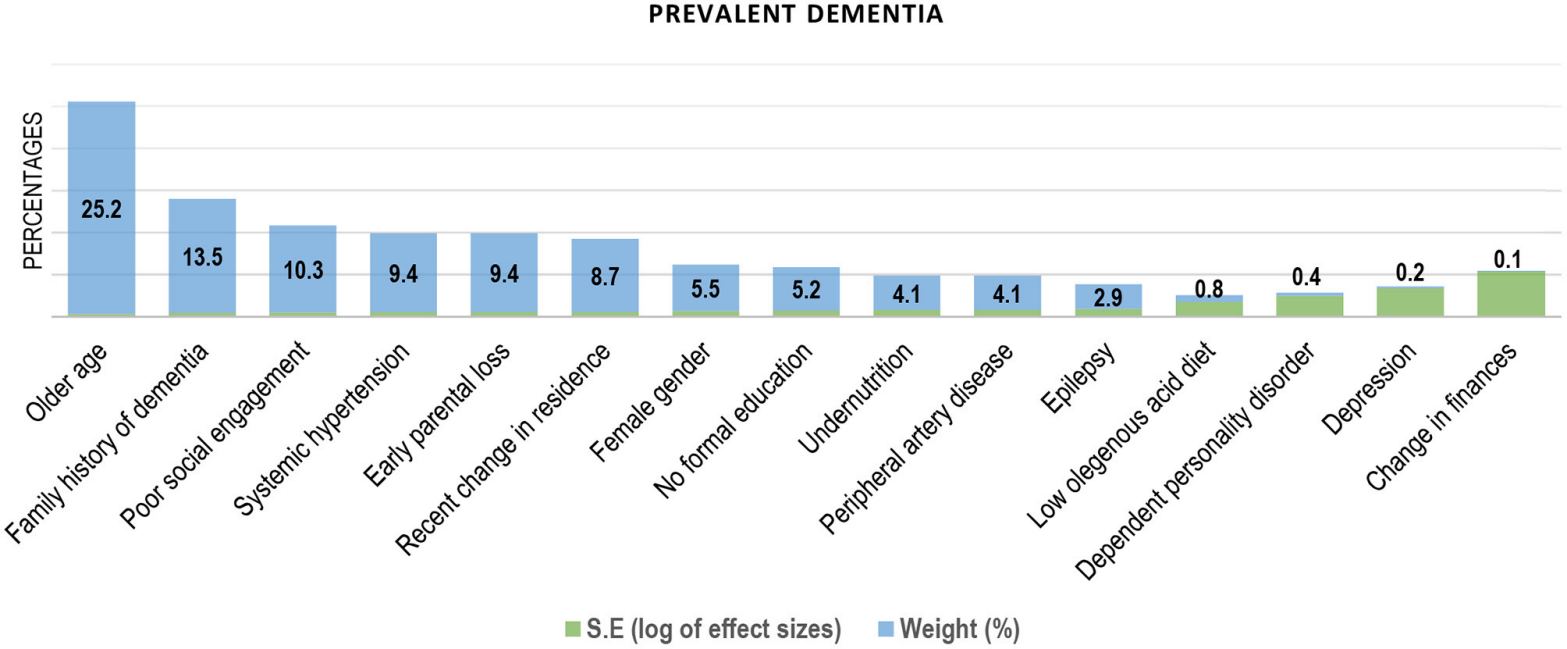
Factors associated with prevalent dementia in Sub-Saharan Africa (ranked by the inverse of variance method).

**Figure 4:**
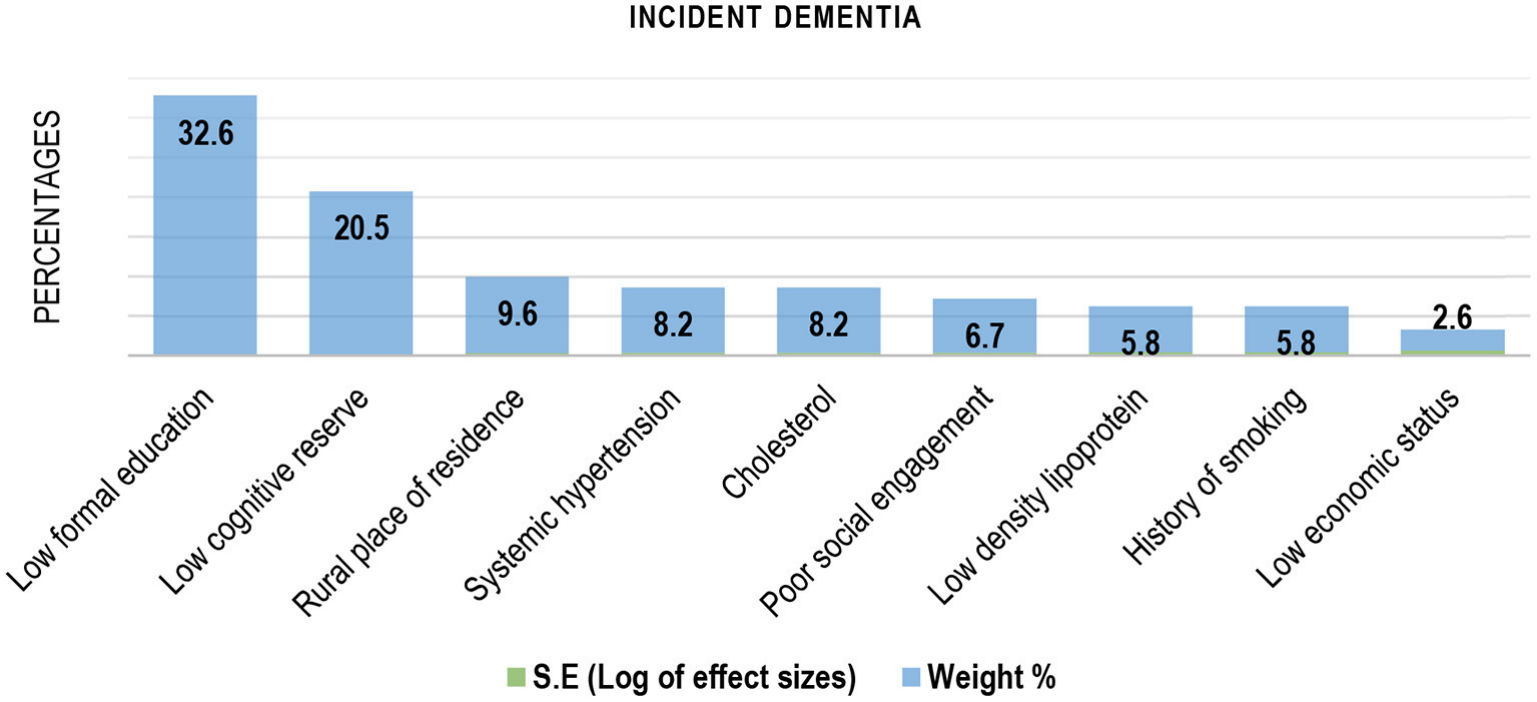
Modifiable risk factors for Incident dementia in Sub-Saharan Africa (ranked by the inverse of variance method).

Older age was the most cited and independent factor associated with prevalent dementia (31, 34, 35, 39–41, 50–53) in SSA (Figure 3). Older age was also the dominant risk factor for incident dementia in the sub-region (Table 2). Figure 4 contains the pooled modifiable risk factors for incident dementia in SSA ranked according to estimates of S.Es of their independent association with incident dementia. The strongest evidence on modifiable risk factors is the association of low educational attainment and poor pre-dementia cognitive functioning (cognitive reserve) with incident dementia. The association of vascular and other social risk factors was less precise by demonstrating large S.Es (Figure 4).

**Table 2:** Independent risk factors for incident dementia in Sub-Saharan Africa (ranked by the inverse of variance method).

**Independently associated factors**	**Standard errors**	**Weight %**
Age	0.02	95.8
Low formal education	0.19	1.1
Poor predementia cognitive functioning	0.24	0.7
Apolipoprotein E4 homozygosity	0.29	0.5
Rural place of residence	0.35	0.3
Female gender	0.37	0.3
Systemic hypertension	0.38	0.3
Cholesterol	0.38	0.3
Poor social engagement	0.42	0.2
Low density lipoprotein	0.45	0.2
History of smoking	0.45	0.2
Low occupational attainment	0.46	0.2
Low economic status	0.67	0.1

#### Publication Bias and Sensitivity Analysis

The funnel plot in Supplementary Figure 1 showed no clear evidence of publication bias. Sensitivity analyses conducted according to geographical location of studies suggest that studies from Nigeria reported distinctly low rates of dementia compared with studies conducted in other parts of SSA (Supplementary Figure 2).

## Discussion

The pooled annual incidence of dementia in SSA is ≈2%, while the pooled prevalence is ≈5 and 9%, respectively, when diagnosed using clinical assessment criteria and rating scales. Age was the dominant risk factor for both prevalent and incident dementia, while low educational attainment and poor pre-dementia cognitive functioning were the prominent modifiable risk factors for incident dementia in SSA.

Our findings overlap with pooled global estimates (54) of dementia prevalence, incidence and dominant risk factors as well as estimates derived from other low- or middle-income countries (LMICs) (54, 55). Notably, there is still a significant gap in the literature on the pooled incidence of dementia from across LMICs to which our findings could be compared (54). Our current estimate of 5% prevalence of dementia is higher than our previous rate of 4% (3) because of the inclusion of data from six additional studies: two from South Africa and one each from Congo, Central African Republic, Tanzania, and Nigeria. The estimated 2% annual incidence of dementia in the present study is also higher than the 1.3% estimated previously (2, 4). These increases in rates may suggest greater awareness of dementia in the sub-region since 2016 or, otherwise, more people may now be living with dementia in SSA compared to when pooled estimates of dementia incidence and prevalence were last conducted. An increase in rates of dementia over time could be expected as it is in keeping with the phenomenon of global population aging and the projected increase in the number of older people living with dementia in SSA and other LMIC contexts (1).

The inclusion of six additional studies estimating prevalence of dementia in five SSA countries thus meant that our pooled estimate of prevalence is likely more reflective of the occurrence of dementia in the sub-region. However, some of the risk factors identified from cross-sectional studies may be prone to the effect of reverse causality. This effect may have resulted in larger sizes of association between dementia and, for example, poor social engagement or recent change in residence (as would be expected for placement in long term institutional care). Conversely, the impact of factors such as depression, undernutrition and changes in finances, which may be increasingly associated with dementia overtime may be under-estimated in cross-sectional investigations. This is because cross-sectional analyses are inadequate in providing robust evidence for the direction of association between relevant health conditions overtime.

Our meta-analysis of modifiable risk factors for incident dementia included five studies. Previous systematic reviews of incidence of dementia in SSA have relied on two (2) or four studies (4). We were able to identify one additional study estimating the incidence of dementia in rural and urban Congo after a follow-up period of 2 years. Unlike our estimate of prevalence, the annual incidence of dementia reported in the present study is unlikely to be generalizable to all SSA regions. This is partly because 45.2% of the evidence is from one country, Nigeria. A sensitivity analyses conducted by geographical location of identified studies showed that studies from Nigeria reported distinctly low rates of dementia relative to studies conducted in other parts of SSA. This would suggest that the relatively large numbers of studies from Nigeria could have led to an underestimation, rather than overestimation, of the true rates of dementia in SSA. Even though our funnel plot showed no clear evidence of publication bias, the observation that many African studies are published in less visible or less accessible media could also have affected our pooled estimates. We note that our search strategy included the African journals online database. However, our failure to incorporate gray literature in our searches would mean that a few studies may have been missed, and their results not included in our meta-analyses.

Most of the primary citations identified for the present systematic review did not report rates of dementia according to relevant age groups and sex. As such, our reported estimates are not age or gender standardized. This methodological limitation could, in part, have accounted for the differences in rates reported in the present study and those reporting age and gender standardized rates (2). Variations in pooled rates of dementia have also been previously reported to reflect the use of different dementia-ascertainment procedures (56). In the present systematic review, we have combined data comprising similar diagnostic procedures in the same meta-analysis model. Whereas, previous estimates had been based on data pooled from studies regardless of dementia ascertainment procedures.

Our findings in relations to risk factors for dementia in the present study were not surprising. Life course higher educational attainment and pre-dementia cognitive functioning have been demonstrated as indices of biological (57) and socio-economic (58) protection against the neuro-degenerative changes that may result in dementia in older people. This phenomenon is often viewed as being indicative of cognitive reserve (59). Similar to reports from higher income countries (56–58), these proxy indicators of cognitive reserve also appear to have important association with incident dementia in SSA.

In SSA, low formal educational attainment in particular may be considered as a stable index of low economic status over the life course (60). In most of SSA were there is a steady socio-economic differential in health across the lifespan (60), the disadvantage of belonging in a low economic status may accumulate over the life-course (61). This accumulation may, in turn, translate to significant risks to health, including the possibility of dementia by the age of 65 years (62). We note that educational attainment was assessed in the reviewed studies as either the number of years of formal education completed or whether participants attended primary, secondary or higher education. On the other hand, pre-dementia cognitive functioning was defined by the performance of participants on the learning phase of the 10-word listing test (10-WDRT). Scores on this test were dichotomised as “poor,” for dementia free participants who scored <1 standard deviation (SD) below the mean score for 3 administrations of the 10-WDRT, and good for the other dementia free respondents (63).

### Research and Clinical Implications

In line with the phenomenon of socio-economic differential in health, individuals surviving to old age in most of SSA, where life expectancy at birth is relatively low (64), may include a comparatively healthier section of the population. This group may also have a lower latent risk of dementia while those with higher cumulative morbidity may be more likely to die at a younger ages (60). In a country like Nigeria, as an example, it is projected that despite an average life expectancy at birth of about 52 years (65), the population surviving to the age of 65 years may have the prospect of an additional 15 years of life (66, 67). It is important to note that Nigeria also provided about 50% of the studies included in the present review.

Despite biases related sample size which was partly due to several studies reporting from the same cohort, our meta-analysis makes several additions to the literature on the epidemiology of dementia in SSA. First, the addition of six new studies published in the last 4 years and a half resulted in some increase in sample size, as well as the possibility of greater precision and generalization of our findings to most of SSA. Second, we were able to conduct sub-group analyses demonstrating that pooled rates of dementia are higher when combining studies using rating scales ascertainment. Whereas, hospital-based studies as well as those using clinical diagnostic criteria report lower rates. The low frequency of dementia found in hospital-based studies included in the present systematic review may reflect a possible low healthcare utilization which may also result from prevailing sociocultural practices and pathways to care (68).

### Conclusion

The estimated pooled annual incidence of clinically diagnosed dementia in SSA is ≈2%, and the prevalence is ≈5%. Estimated rates vary according to dementia assessment procedures and types of study populations. As reported globally, older age was the dominant risk factor for dementia in the present study, while low educational attainment was the most prominent modifiable factor. The present study adds to the literature on the epidemiology of dementia in SSA by generating potentially more precise and generalizable estimates due to larger sample size. The findings have implications for deliberate policies targeted at access to education across the life course as a primary prevention strategy against dementia in SSA.

## Data Availability Statement

The original contributions presented in the study are included in the article/Supplementary Material, further inquiries can be directed to the corresponding author/s.

## Author Contributions

AO conceived and designed the study. Material preparation, data collection and analysis were performed by AO and APO. The first draft of the manuscript was written by AO and OB. All authors read and approved the final draft.

## Conflict of Interest

The authors declare that the research was conducted in the absence of any commercial or financial relationships that could be construed as a potential conflict of interest.
