# Supplementary material for: Dominant and Modifiable Risk Factors for Dementia in Sub-Saharan Africa: A Systematic Review and Meta-Analysis
Source: Front Neurol. 2021 Mar 25;12:627761. doi: 10.3389/fneur.2021.627761 (PMC8027065; doi:10.3389/fneur.2021.627761)

Supplementary Figure 1: Funnel plot for the epidemiology of dementia in sub-Saharan Africa

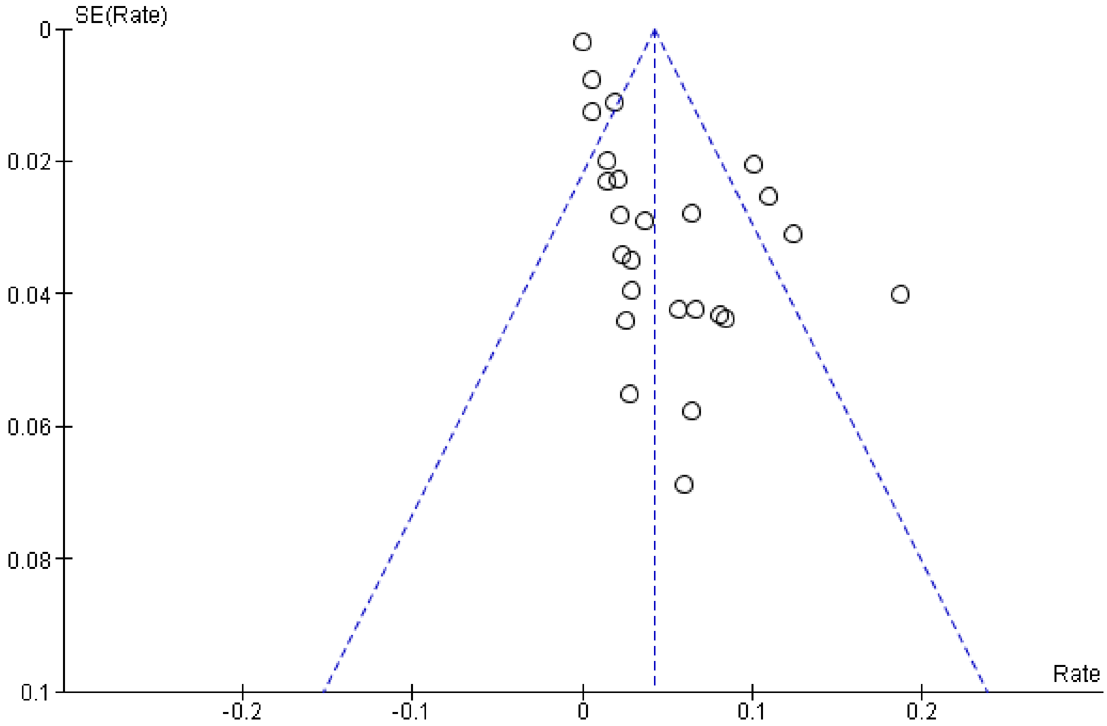

Supplementary Figure 2: Sensitivity analyses

Prevalence reported from Nigeria

Study or Subgroup	Rate	SE	Weight	Rate IV, Random, 95% CI
Amoo et al 2011	0.0005	0.002	28.4%	0.00 [-0.00, 0.00]
Calliste et al 2015	0.124	0.031	0.0%	0.12 [0.06, 0.18]
De Jager et al 2017	0.11	0.0254	0.0%	0.11 [0.06, 0.16]
Gao et al 2016	0.014	0.023	0.0%	0.01 [-0.03, 0.06]
Guerchet et al 2009	0.026	0.0441	0.0%	0.03 [-0.06, 0.11]
Guerchet et al 2010 (Central African Republic)	0.081	0.0431	0.0%	0.08 [-0.00, 0.17]
Guerchet et al 2010 (Congo)	0.067	0.0423	0.0%	0.07 [-0.02, 0.15]
Guerchet et al 2017 (Central African Republic)	0.084	0.0439	0.0%	0.08 [-0.00, 0.17]
Guerchet et al 2017 (Congo)	0.057	0.0423	0.0%	0.06 [-0.03, 0.14]
Gureje et al 2006	0.101	0.0205	20.6%	0.10 [0.06, 0.14]
Gureje et al 2011	0.022	0.0282	0.0%	0.02 [-0.03, 0.08]
Hendrie et al 2001	0.014	0.0201	0.0%	0.01 [-0.03, 0.05]
Napon et al 2009	0.005	0.0079	0.0%	0.01 [-0.01, 0.02]
Oango et al 2014	0.019	0.0111	0.0%	0.02 [-0.00, 0.04]
Ochayi et al 2006	0.064	0.0577	7.0%	0.06 [-0.05, 0.18]
Ogunniyi et al 1993	0.006	0.0127	24.8%	0.01 [-0.02, 0.03]
Ogunniyi et al 2016	0.029	0.0396	11.7%	0.03 [-0.05, 0.11]
Ojagbemi et al 2016	0.021	0.0227	0.0%	0.02 [-0.02, 0.07]
Paddick et al 2013	0.064	0.0279	0.0%	0.06 [0.01, 0.12]
Paddick et al 2018	0.187	0.04	0.0%	0.19 [0.11, 0.27]
Paraiso et al 2011	0.037	0.029	0.0%	0.04 [-0.02, 0.09]
Samba 2015	0.0234	0.0341	0.0%	0.02 [-0.04, 0.09]
Siddiqi et al 2010	0.029	0.035	0.0%	0.03 [-0.04, 0.10]
Vandepoel et al 2013	0.06	0.0686	0.0%	0.06 [-0.07, 0.19]
Yusuf et al 2010	0.028	0.055	7.5%	0.03 [-0.08, 0.14]
**Total (95% CI)**			**100.0%**	**0.03 [-0.00, 0.07]**

Heterogeneity: Tau² = 0.00; Chi² = 25.81, df = 5 (P < 0.0001); I² = 81%
Test for overall effect: Z = 1.84 (P = 0.07)

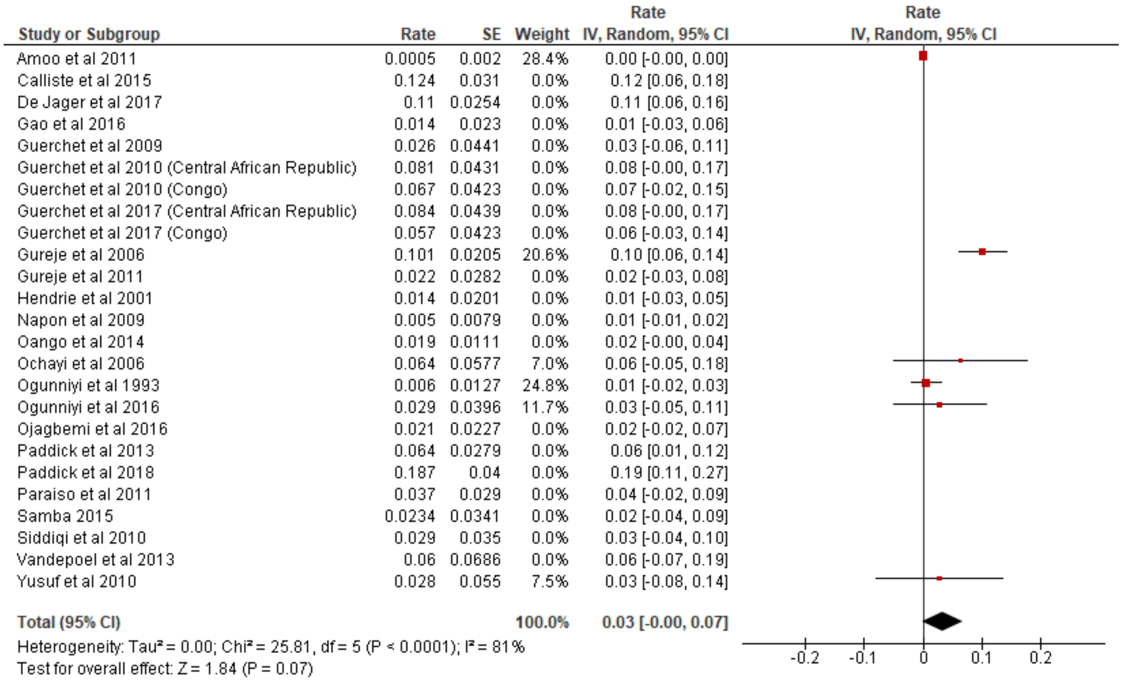

Prevalence reported from rest of sub-Saharan Africa

Study or Subgroup	Rate	SE	Weight	Rate IV, Random, 95% CI
Amoo et al 2011	0.0005	0.002	0.0%	0.00 [-0.00, 0.00]
Calliste et al 2015	0.124	0.031	7.6%	0.12 [0.06, 0.18]
De Jager et al 2017	0.11	0.0254	8.7%	0.11 [0.06, 0.16]
Gao et al 2016	0.014	0.023	0.0%	0.01 [-0.03, 0.06]
Guerchet et al 2009	0.026	0.0441	5.5%	0.03 [-0.06, 0.11]
Guerchet et al 2010 (Central African Republic)	0.081	0.0431	5.7%	0.08 [-0.00, 0.17]
Guerchet et al 2010 (Congo)	0.067	0.0423	5.8%	0.07 [-0.02, 0.15]
Guerchet et al 2017 (Central African Republic)	0.084	0.0439	5.6%	0.08 [-0.00, 0.17]
Guerchet et al 2017 (Congo)	0.057	0.0423	5.8%	0.06 [-0.03, 0.14]
Gureje et al 2006	0.101	0.0205	0.0%	0.10 [0.06, 0.14]
Gureje et al 2011	0.022	0.0282	0.0%	0.02 [-0.03, 0.08]
Hendrie et al 2001	0.014	0.0201	0.0%	0.01 [-0.03, 0.05]
Napon et al 2009	0.005	0.0079	11.7%	0.01 [-0.01, 0.02]
Oango et al 2014	0.019	0.0111	11.3%	0.02 [-0.00, 0.04]
Ochayi et al 2006	0.064	0.0577	0.0%	0.06 [-0.05, 0.18]
Ogunniyi et al 1993	0.006	0.0127	0.0%	0.01 [-0.02, 0.03]
Ogunniyi et al 2016	0.029	0.0396	0.0%	0.03 [-0.05, 0.11]
Ojagbemi et al 2016	0.021	0.0227	0.0%	0.02 [-0.02, 0.07]
Paddick et al 2013	0.064	0.0279	8.2%	0.06 [0.01, 0.12]
Paddick et al 2018	0.187	0.04	6.1%	0.19 [0.11, 0.27]
Paraiso et al 2011	0.037	0.029	8.0%	0.04 [-0.02, 0.09]
Samba 2015	0.0234	0.0341	0.0%	0.02 [-0.04, 0.09]
Siddiqi et al 2010	0.029	0.035	6.9%	0.03 [-0.04, 0.10]
Vandepoel et al 2013	0.06	0.0686	3.1%	0.06 [-0.07, 0.19]
Yusuf et al 2010	0.028	0.055	0.0%	0.03 [-0.08, 0.14]
**Total (95% CI)**			**100.0%**	**0.06 [0.04, 0.09]**

Heterogeneity: Tau² = 0.00; Chi² = 51.33, df = 13 (P < 0.00001); I² = 75%
Test for overall effect: Z = 4.50 (P < 0.00001)

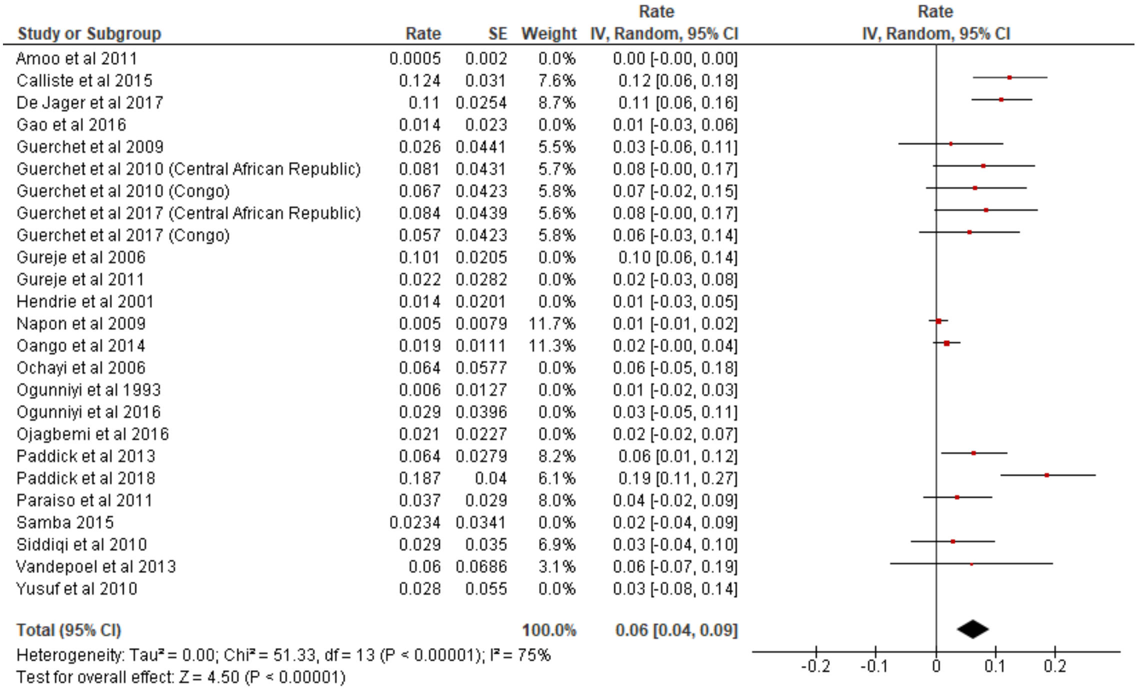

Supplement: Supplementary file 1 [file Image_1.pdf]
